# *Septin5* deficiency impairs both recent and remote contextual fear memory

**DOI:** 10.1186/s13041-025-01260-4

**Published:** 2025-11-13

**Authors:** Natsumi Ageta-Ishihara, Naoto Fukumasu, Kodai Sakakibara, Kazuki Fujii, Yumie Koshidaka, Saori Katsuragawa, Kenji Tanigaki, Takeshi Hiramoto, Gina Kang, Noboru Hiroi, Yugo Fukazawa, Tsuyoshi Miyakawa, Keizo Takao, Makoto Kinoshita

**Affiliations:** 1 Department of Biomolecular Science, Faculty of Science, Toho University, 2-2-1 Miyama, Funabashi, Chiba 274-8510 Japan; 2 Department of Molecular Biology, Division of Biological Sciences, Nagoya University Graduate School of Science, Nagoya, Japan; 3 Department of Behavioral Physiology, Faculty of Medicine, University of Toyama, Toyama, Japan; 4 Life Science Research Center, University of Toyama, Toyama, Japan; 5 Research Institute, Shiga Medical Center, Moriyama, Shiga, Japan; 6 Clinical Research Center, Shiga General Hospital, Moriyama, Shiga Japan; 7 Department of Pharmacology, University of Texas Health Science Center at San Antonio, San Antonio, USA; 8 Department of Cellular and Integrative Physiology, University of Texas Health Science Center at San Antonio, San Antonio, USA; 9 Department of Cell Systems and Anatomy, University of Texas Health Science Center at San Antonio, San Antonio, USA; 10 Division of Brain Structure and Function, Faculty of Medical Science, University of Fukui, Fukui, Japan; 11 Division of Systems Medical Science, Center for Medical Science, Fujita Health University, Aichi, Japan; 12 Center for Genetic Analysis of Behavior, National Institute for Physiological Sciences, Okazaki, Aichi Japan

**Keywords:** Septins, *Septin5*, Hippocampus, Contextual fear memory

## Abstract

Septin-3 and Septin-5 are components of the septin cytoskeleton highly expressed in the nervous system, yet the extent of their shared and distinct roles is not fully understood. We recently demonstrated that Septin-3 regulates late-phase long-term potentiation (L-LTP)-dependent invasion of smooth endoplasmic reticulum (sER) into dentate gyrus (DG) spines. *Septin3*^−/−^ mice exhibit normal synaptic ultrastructure in the hippocampal DG, CA3, and CA1, yet the fraction of sER-containing spines is reduced; behaviorally, they show selective deficits in 1-day object recognition and 1-day contextual fear memory, whereas cued fear conditioning and contextual memory tested at 1 month are intact. Here, using adult male *Septin5*^−/−^ mice, we tested whether morphological and behavioral phenotypes identified in *Septin3*^−/−^ mice are shared or subunit-specific. Electron microscopy showed no detectable differences in synapse density, spine volume, and postsynaptic density (PSD) area in the hippocampal DG, CA3, and CA1, with an unchanged fraction of sER-containing spines relative to wild-type littermates. Behaviorally, *Septin5*^−/−^ mice were impaired in recent (1 day) and remote (1 month) contextual fear memory, but were normal in 1-day novel object recognition memory and in recent and remote cued fear memory. The shared and distinct structural and behavioral phenotypes observed in *Septin5*^−/−^ and *Septin3*^−/−^ mice suggest either sER-independent common mechanisms or subunit-specific ones for recent contextual fear memory deficit, and indicate a Septin-5-dependent contribution to remote contextual fear memory.

**Supplementary Information:**

The online version contains supplementary material available at 10.1186/s13041-025-01260-4.

## Main text

Septins are cytoskeletal filament-forming GTPases that assemble into hetero-oligomers and higher-order filaments [1]. In mammals, the septin family comprises Septin-1– Septin-12 and Septin-14 (Septin-13 is a pseudogene) [2]. Among them, Septin-3/G-septin and Septin-5/CDCrel-1 are highly expressed in the nervous system [3–5]. In the hippocampal CA1 region, the overall architecture is normal in *Septin3*^−/−^, *Septin5*^−/−^, and *Septin3*^−/−^ *Septin5*^−/−^ mice [6]. Electrophysiological studies further indicate that synaptic properties at CA1 synapses remain intact in *Septin5*^−/−^ and *Septin3*^−/−^ *Septin5*^−/−^ mice [6, 7]. Nevertheless, *Septin5*^−/−^ mice show reductions in affiliative social interaction depending on genetic background, whereas decreased anxiety-related behavior on the elevated plus maze, enhanced prepulse inhibition, and slower acquisition in a rewarded approach task have been reported without detectable effects of genetic background [8]. The impaired social interaction in *Septin5*^−/−^ mice is due to a dose alteration of this gene in the hippocampus [9]. We previously showed that *Septin3*^−/−^ mice exhibit normal synapse density, spine volume, and PSD area, whereas the fraction of sER-containing spines is reduced in the hippocampal DG as well as in CA3 and CA1; L-LTP drives sER entry into DG spines in a Septin-3-dependent manner. Basal transmission at perforant path–DG synapses is unchanged, yet *Septin3*^−/−^ mice exhibit selective 1-day deficits in object recognition and contextual fear memory, whereas cued fear and 1-month contextual memory remain intact [10–12]. It remains unclear whether Septin-5 and Septin-3 exhibit similar or distinct phenotypes in these measures. To address this, we examined synaptic ultrastructure in the hippocampal DG, CA3, and CA1 and assessed 1-day object recognition as well as contextual and cued fear memory at 1 day and 1 month in *Septin5*^−/−^ mice.

**Fig. 1:**
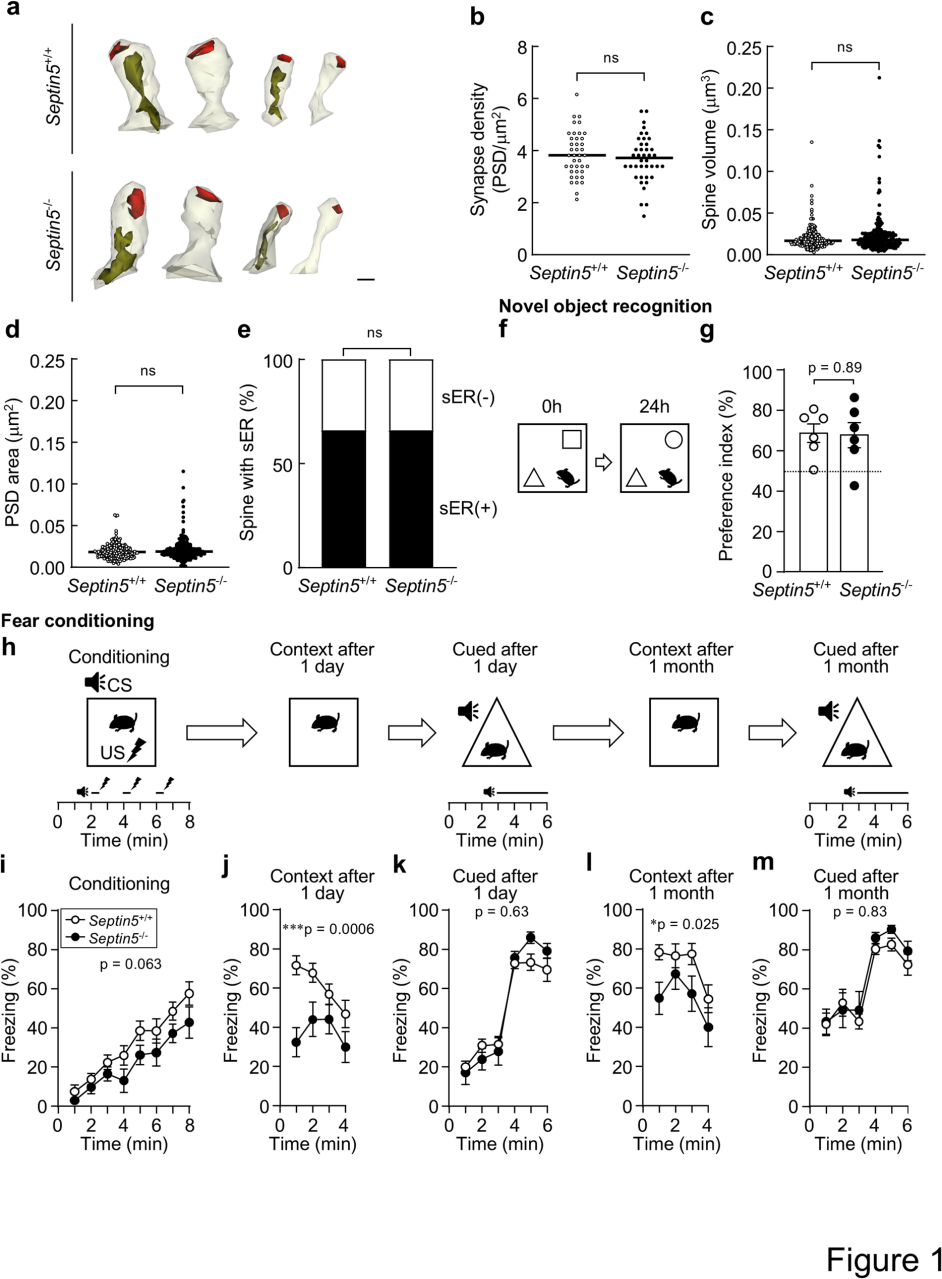
Septin-5 deficiency impairs contextual fear memory while sparing DG ultrastructure and 1-day object recognition**. a**, Representative 3D reconstructions of dendritic spines with or without sER (green) and PSD area (red) in the middle molecular layer of the DG of 10-week-old male *Septin5*^+/+^ and *Septin5*^−/−^ mice, reconstructed from serial-section transmission electron microscopy. Scale bar, 100 nm. **b**, Synapse density. n = 40 dissector pairs of sections taken from four areas across both hemispheres in the DG middle molecular layer in two 10-week-old littermate male *Septin5*^+/+^ and *Septin5*^−/−^ mice; Mann–Whitney test. Data are shown as median. **c**, Spine volume. n =  205 (*Septin5*^+/+^) and 208 (*Septin5*^−/−^) spines; Mann–Whitney test. Data are shown as median. **d**, PSD area. n =  204 (*Septin5*^+/+^) and 206 (*Septin5*^−/−^) spines; Mann–Whitney test. Data are shown as median. **e**, Percentage of spines containing smooth endoplasmic reticulum (sER). n =  191 (*Septin5*^+/+^) and 200 (*Septin5*^−/−^) spines; Fisher’s exact test. Data are shown as median (**b**–**d**). ns, not significant. **f**, **g**, Novel object recognition test. **f**, Schematic of novel object recognition test. **g**, Preference index 1 day after training, calculated as 100 × [exploration time of the novel object]/[sum of exploration times for the novel and familiar objects]; the dashed line indicates chance level (50%). n =  6 (*Septin5*^+/+^) and 6 (*Septin5*^−/−^) 20–21-week-old male mice; two-tailed unpaired *t* test. Data are mean ±  SEM. ns, not significant. **h–m**, Contextual and cued fear conditioning test. **h**, Schematic of contextual and cued fear conditioning test. **i**, Freezing during acquisition of the association between a foot shock and a preceding auditory cue (tone) in square chamber [genotype main effect, F_1,30_ =  4.07, *p* =  0.053, genotype  ×  time interaction, F_7,210_ =  0.51, *p* =  0.83]. **j**, **l**, Freezing in square (context) chamber without the cue, tested 1 day (**j**) or 1 month (**l**) after conditioning [1 day, genotype main effect, F_1,30_ =  14.79, *p* =  0.0006, genotype × time interaction, F_3,90_ = 1.96, *p* =  0.13, 1 month, genotype main effect, F_1,30_ =  5.60, *p* = 0.025, genotype × time interaction, F_3,90_ = 0.60, *p* = 0.62]. **k**, **m**, Freezing in response to the tone in triangular (cued) chamber, tested 1 day (**k**) or 1 month (**m**) after conditioning [1 day, genotype main effect, F_1,30_ =  0.24, *p* =  0.63, genotype × time interaction, F_5,150_ = 2.02, *p* = 0.079, 1 month, genotype main effect, F_1,30_ = 0.61, *p* = 0.44, genotype × time interaction, F_5,150_ = 0.42, *p* = 0.83]. n = 19 (*Septin5*^+/+^) and n =  13 (*Septin5*^−/−^) 28–35-week-old (**i**–**k**) or 32–39-week-old (**l**,** m**) male mice; mixed-effects model (REML) with time (minutes) as a within-subject repeated factor (subject = mouse) and fixed effects of genotype, time, and genotype  ×  time; graphical diagnostics (residual, homoscedasticity, and QQ plots) did not reveal evidence of major assumption violations. Data are mean ± SEM. *p <  0.05, ***p <  0.001

To examine DG morphology in *Septin5*^−/−^ mice, we performed serial-section transmission electron microscopy in the middle molecular layer, as in our *Septin3*^−/−^ study [11]. Synapse density, spine volume, and PSD area of asymmetric (predominantly glutamatergic) synapses were indistinguishable between *Septin5*^−/−^ and wild-type (*Septin5*^+/+^) mice (Fig. 1a–d), and—unlike *Septin3*^−/−^ mice—the fraction of sER-containing spines was unchanged (Fig. 1e). Synaptic ultrastructure in CA3 and CA1 showed no detectable differences (Fig. S1). These ultrastructural measurements indicate that hippocampal spine architecture is preserved in the absence of Septin-5.

We next evaluated 1-day object recognition in the novel object recognition task (Fig. 1f). The preference index at 1 day was comparable between *Septin5*^−/−^ and wild-type mice (Fig. 1g). These data indicate that 1-day object recognition is preserved in *Septin5*^−/−^ mice.

Motivated by our prior finding that *Septin3*^−/−^ mice are impaired in 1-day contextual but not 1-month contextual or cued fear memory, we evaluated both paradigms in *Septin5*^−/−^ mice (Fig. 1h). During acquisition in the square chamber, freezing increased over time in both *Septin5*^+/+^ and *Septin5*^−/−^ mice; the difference in how freezing changed over time between *Septin5*^+/+^ and *Septin5*^−/−^ mice approached, but did not reach, statistical significance (mixed-effects model, Fig. 1i). During each footshock, locomotor responses were comparable between *Septin5*^+/+^ and *Septin5*^−/−^ mice (Fig. S2), arguing against altered shock perception or gross locomotor differences. In the same cohort, light/dark transition test data were comparable between *Septin5*^+/+^ and *Septin5*^−/−^ mice (Fig. S3), indicating that baseline anxiety-like behavior, as assessed by this test, was similar in both *Septin5*^+/+^ and *Septin5*^−/−^ mice. These observations are directionally consistent with prior work in C57BL/6J cohorts showing that *Septin5*^+/−^ and *Septin5*^−/−^ mice—as well as mice with region-specific overexpression in the dorsal hippocampus or amygdala—showed no discernible differences relative to *Septin5*^+/+^ mice in motor or anxiety-related behaviors on the open field and elevated plus maze tests [9], and with findings that *Septin5*^−/−^ mice did not alter startle behavior on mixed and 129-enriched backgrounds [8]. Contextual memory, probed in the square (context) chamber without the auditory cue, was reduced in *Septin5*^−/−^ mice at 1 day (Fig. 1j) and this reduction persisted at 1 month after conditioning (Fig. 1l). By contrast, cued memory in the triangular chamber was comparable between *Septin5*^+/+^ and *Septin5*^−/−^ mice at 1 day (Fig. 1k) and at 1 month after conditioning (Fig. 1m). Together, although a subtle difference during conditioning cannot be excluded, these results indicate that *Septin5* deficiency selectively impairs 1-day contextual memory with persistence at 1 month, while cued memory remains intact.

Collectively, our comparative analysis indicates that Septin-5 is dispensable for hippocampal ultrastructure—including an unchanged fraction of sER-containing spines—and that Septin-5 deficiency selectively impairs 1-day contextual fear memory with persistence at 1 month, while cued memory remains intact. Given that both contextual fear conditioning and social interaction are mediated, at least in part, by the hippocampus [13, 14], the latter observation is consistent with our previous report that virally-guided over-expression of *Septin5* in the hippocampus elevates active social interaction [9]. On the other hand, *Septin5* gene-dose alterations appear to differentially impact amygdala-dependent tasks. Its overexpression in the amygdala elevates social behavior [9], whereas *Septin5*^−/−^ mice were normal in cued fear memory—a task that is predominantly amygdala-dependent and not typically dependent on the hippocampus [15–18]—consistent with task- and circuit-specific effects rather than generalized amygdala dysfunction. At the same time, given that Septin-5 is expressed beyond the hippocampus [19], it will be important in future work to perform region-specific rescue experiments—for example, virally guided over-expression of Septin-5 in hippocampal and/or candidate extra-hippocampal circuits—to determine whether the contextual memory phenotype reflects hippocampal and/or non-hippocampal components. Once a contributory non-hippocampal circuit is implicated, region-specific ultrastructural analyses will be carried out to test for synaptic correlates of the phenotype in that area. These findings suggest either sER-independent common mechanisms or subunit-specific mechanisms for the 1-day contextual fear memory deficit, with the role of sER remaining uncertain; they also suggest a Septin-5-specific contribution to remote contextual memory at 1 month.

Consistent with sER-independent mechanisms, Septin-5 interacts with the presynaptic SNARE (soluble *N*-ethylmaleimide-sensitive fusion protein attachment protein receptor) machinery: it binds syntaxin-1 and can compete with α-SNAP (soluble *N*-ethylmaleimide-sensitive factor attachment protein) for SNARE-complex binding, suggesting a route to altered transmitter release [5, 20]. Moreover, Cdk5 (cyclin-dependent kinase 5)-dependent phosphorylation of Septin-5 decreases the Septin-5–syntaxin-1 interaction, indicating regulation of this interface [21]. In line with subunit-specific mechanisms, Septin-5 harbors a putative C-terminal coiled-coil region, whereas Septin-3 lacks this segment [22], implying interacting partners and assemblies. Accordingly, brain region–specific, unbiased identification of interacting partners of Septin-5 and Septin-3 may clarify their shared and distinct mechanisms at the molecular level.

## Methods

Methods are described in the Supplementary Materials.

## Supplementary Information

Below is the link to the electronic supplementary material.

Supplementary Material 1

## Data Availability

The datasets generated and analyzed during the current study are available from the corresponding author on reasonable request.

## Abbreviations

L-LTP: Late-phase long-term potentiation
sER: Smooth endoplasmic reticulum
DG: Dentate gyrus
PSD: Postsynaptic density
SNARE: Soluble *N*-ethylmaleimide-sensitive fusion protein attachment protein receptor
α-SNAP: Soluble *N*-ethylmaleimide-sensitive factor attachment protein
Cdk5: Cyclin-dependent kinase 5
