# Supplementary material for: Septin5 deficiency impairs both recent and remote contextual fear memory
Source: Mol Brain. 2025 Nov 13;18:85. doi: 10.1186/s13041-025-01260-4 (PMC12613764; doi:10.1186/s13041-025-01260-4)
Supplement: Supplementary file 1 — Supplementary Material 1 [file 13041_2025_1260_MOESM1_ESM.pdf]

Supplementary Materials

Methods

Animals

All animal experiments were approved by the Institutional Animal Care and Use Committees of Fujita Health University, the National Institute for Physiological Sciences, University of Toyama, and Nagoya University, and were carried out in accordance with institutional guidelines for the care and use of laboratory animals. Animals were housed under a 12-h light/dark cycle with food and water available ad libitum.

Septin5 knockout (*Septin5*$^{-/-}$) mice were generated by backcrossing the original *Septin5* mutant line [1] to C57BL/6J mice for more than 10 generations [2] to control for the impact of the genetic background [3]. Subsequently, this line was backcrossed to C57BL/6N mice for more than 10 generations. *Septin5*$^{-/-}$ and wild-type (*Septin5*$^{+/+}$) littermates were obtained by mating *Septin5*$^{+/-}$ heterozygous mice or by *in vitro* fertilization using gametes from *Septin5*$^{+/-}$ mice. Male *Septin5*$^{-/-}$ mice and wild-type controls were used for experiments, and the specific ages at testing are indicated in the figure legends.

Electron microscopy

Electron microscopy was performed following established procedures [4, 5]. Briefly, 50-nm ultrathin sections were cut from the middle one-third of the dentate gyrus molecular layer (DG-MML), the stratum radiatum of CA3, and the central stratum radiatum of CA1 in male *Septin5*$^{-/-}$ and *Septin5*$^{+/+}$ littermates. Sections were examined using a transmission electron microscope (JEM-1010; JEOL). Serial images were registered and three-dimensional reconstructions were generated using Reconstruct (SynapseWeb).

For quantification, spine volume, postsynaptic density (PSD) area, and the proportion of spines containing smooth endoplasmic reticulum (sER) were measured by tracing all spines within predefined sampling fields across 30–35 serial ultrathin sections acquired at 25,000× magnification. Synapse density was estimated by the physical dissector method from 30–35 serial sections imaged at 12,000× magnification.

For each animal, four fields per region, combined across both hemispheres, were analyzed in DG-MML, CA3 stratum radiatum, and central CA1 stratum radiatum. All analyses were conducted with the analyst blinded to sample identity. After confirming no significant inter-animal differences within genotype, data were pooled for subsequent analyses.

Behavioral analysis

Novel object recognition test was performed essentially as reported previously [4, 6], with the following parameters. Mice underwent habituation on four consecutive days (6 min/day) consisting of handling on the experimenter's palm followed by free exploration of the test arena (25 × 29 × 29 cm). On the next day, two objects—selected from a sphere (7 × 6.3 cm), an octagonal pyramid (4.6 × 7.4 cm), and a cube (6 × 6 × 6 cm)—were placed in the arena, and mice explored for 15 min. After 24 h, one object was replaced with a novel object and exploration continued for 5 min. Exploration was defined as the mouse orienting toward an object with its nose within 2 cm of the object [7]. The preference index (%) was calculated as 100 × [exploration time of the novel object] / [sum of exploration times for the novel and familiar objects].

Contextual and cued fear conditioning followed established protocols [8, 9]. Briefly, two distinct contexts were used: a square chamber (26 × 34 × 29 cm) with a metal grid floor (100 lux) and a white triangular prism chamber (33 × 29 × 32 cm) (30 lux). On the acquisition day, mice received three presentations of a 30-second white-noise cue, each co-terminating with a mild footshock (0.3 mA). The context test was conducted 1 day (recent) and 1 month (remote) after conditioning in the acquisition context without cues or shocks; following the context test, the cued test was conducted in the alternate context with the cue presented during the latter portion of the session. To assess locomotor responses to footshocks during conditioning, distance traveled (cm) was quantified in 2-second windows immediately before and after each of the three 2-second footshocks (6 seconds per shock). Freezing (immobility time) was quantified automatically.

Light/dark transition test was performed as described previously [8]. Briefly, a two-compartment apparatus (21 × 41 × 25 cm) consisting of connected light and dark chambers was used (390 lux vs. 2 lux). Mice were placed in the dark chamber at the start and allowed to explore for 10 min. Latency to the first entry into the light chamber, time spent in each chamber, number of transitions across the partition, and distance traveled in the light and dark chambers were quantified automatically.

Quantification and statistical analysis

Data are presented as medians or means ± SEM (standard error of the mean). Statistical analyses were performed in GraphPad Prism (GraphPad Software). The statistical tests, P values, sample sizes (n), and definitions of error bars are reported in the figure legends.

References

1. Peng XR, Jia Z, Zhang Y, Ware J, Trimble WS: **The septin CDCrel-1 is dispensable for normal development and neurotransmitter release.** *Mol Cell Biol* 2002, **22:**378-387.
2. Harper KM, Hiramoto T, Tanigaki K, Kang G, Suzuki G, Trimble W, Hiroi N: **Alterations of social interaction through genetic and environmental manipulation of the 22q11.2 gene Sept5 in the mouse brain.** *Hum Mol Genet* 2012, **21:**3489-3499.
3. Hiroi N: **Critical reappraisal of mechanistic links of copy number variants to dimensional constructs of neuropsychiatric disorders in mouse models.** *Psychiatry Clin Neurosci* 2018, **72:**301-321.
4. Ageta-Ishihara N, Fukazawa Y, Arima-Yoshida F, Okuno H, Ishii Y, Takao K, Konno K, Fujishima K, Ageta H, Hioki H, et al: **Septin 3 regulates memory and L-LTP-dependent extension of endoplasmic reticulum into spines.** *Cell Rep* 2025, **44:**115352.
5. Parajuli LK, Ageta-Ishihara N, Ageta H, Fukazawa Y, Kinoshita M: **Methods for immunoblot detection and electron microscopic localization of septin subunits in mammalian nervous systems.** *Methods Cell Biol* 2016, **136:**285-294.
6. Nomoto M, Ohkawa N, Nishizono H, Yokose J, Suzuki A, Matsuo M, Tsujimura S, Takahashi Y, Nagase M, Watabe AM, et al: **Cellular tagging as a neural network mechanism for behavioural tagging.** *Nat Commun* 2016, **7:**12319.
7. Leger M, Quiedeville A, Bouet V, Haelewyn B, Boulouard M, Schumann-Bard P, Freret T: **Object recognition test in mice.** *Nat Protoc* 2013, **8:**2531-2537.
8. Ageta-Ishihara N, Takao K, Miyakawa T, Kinoshita M: **Comprehensive behavioral phenotyping of male Septin 3-deficient mice reveals task-specific abnormalities.** *Mol Brain* 2025, **18:**71.
9. Shoji H, Takao K, Hattori S, Miyakawa T: **Contextual and cued fear conditioning test using a video analyzing system in mice.** *J Vis Exp* 2014.

Supplementary Figures

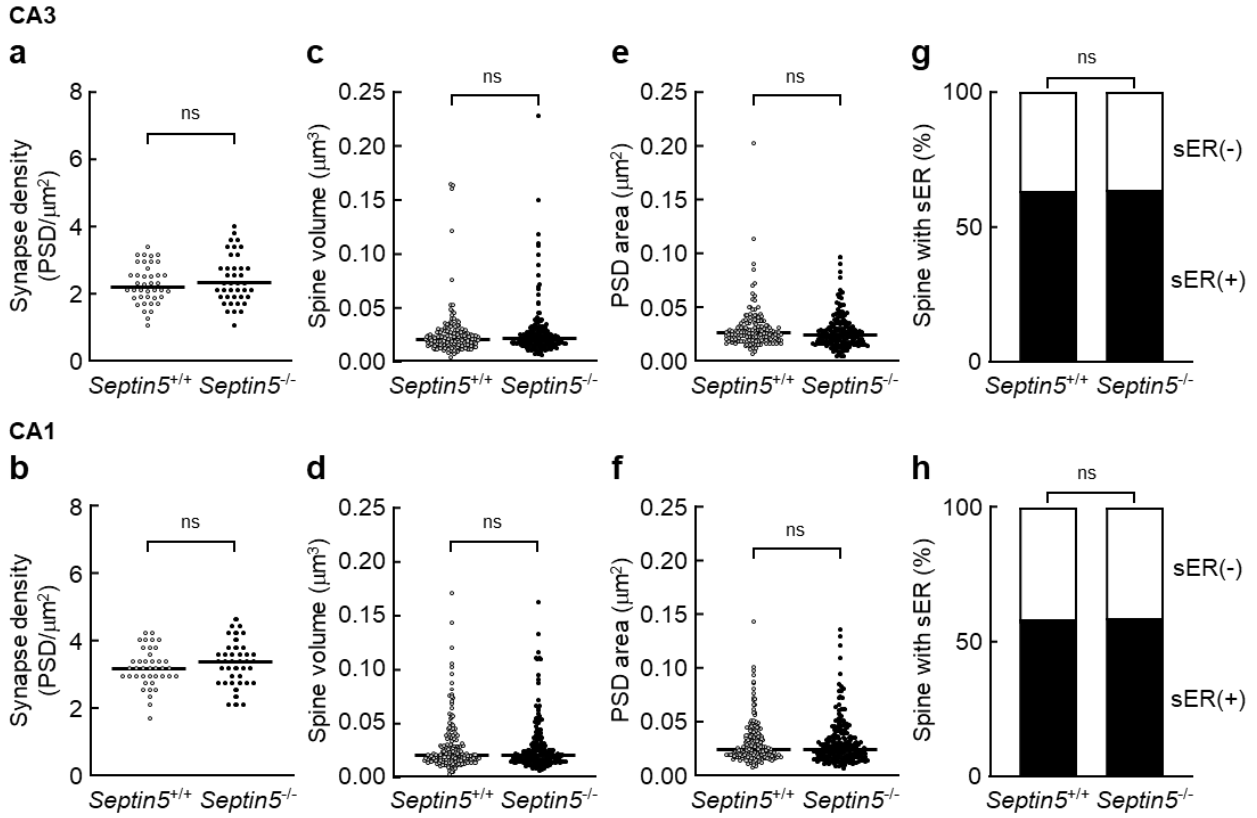

Figure S1

Normal synapse morphology in CA3 and CA1 of *Septin5*$^{-/-}$ mice.

a, **b**, Synapse density in CA3 (**a**) and CA1 (**b**) in 10–19-week-old littermate male *Septin5*$^{+/+}$ and *Septin5*$^{-/-}$ mice. n = 40 dissector pairs of sections from two mice; Mann-Whitney test.

c, **d**, Spine volume. n = 173 (*Septin5*$^{+/+}$) and 181 (*Septin5*$^{-/-}$) spines (CA3), n = 181 (*Septin5*$^{+/+}$) and 206 (*Septin5*$^{-/-}$) spines (CA1); Mann-Whitney test.

e, **f**, PSD area. n = 173 (*Septin5*$^{+/+}$) and 184 (*Septin5*$^{-/-}$) spines (CA3), n = 182 (*Septin5*$^{+/+}$) and 215 (*Septin5*$^{-/-}$) spines (CA1); Mann-Whitney test.

g, **h**, Percentage of spines containing smooth endoplasmic reticulum (sER). n = 173 (*Septin5*$^{+/+}$) and 185 (*Septin5*$^{-/-}$) spines (CA3), n = 181 (*Septin5*$^{+/+}$) and 215 (*Septin5*$^{-/-}$) spines (CA1); Fisher's exact test.

Data are shown as median (**a**–**f**). ns, not significant.

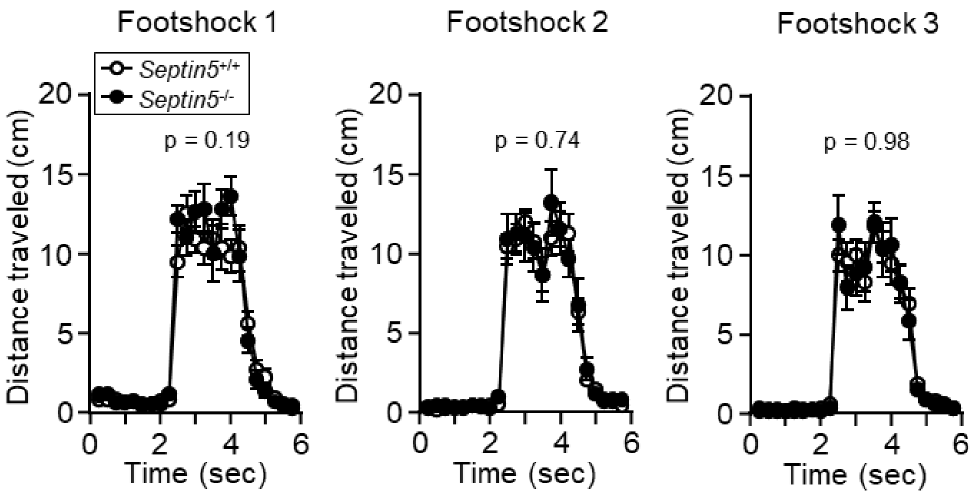

Figure S2

Locomotor responses during footshocks (conditioning).

Distance traveled (cm) in 2-second windows before and after each of the three 2-second footshocks during conditioning. [footshock 1, genotype main effect, $F_{1,30} = 1.78$, $p = 0.19$, genotype × time interaction, $F_{22,660} = 1.39$, $p = 0.11$, footshock 2, genotype main effect, $F_{1,30} = 0.12$, $p = 0.74$, genotype × time interaction, $F_{22,660} = 0.36$, $p = 1.00$, footshock 3, genotype main effect, $F_{1,30} = 0.00044$, $p = 0.98$, genotype × time interaction, $F_{22,660} = 0.41$, $p = 0.99$]. n = 19 (*Septin5*$^{+/+}$) and n = 13 (*Septin5*$^{-/-}$) 28–35-week-old male mice; mixed-effects model (REML) with time (seconds) as a within-subject repeated factor (subject = mouse) and fixed effects of genotype, time, and genotype × time. Data are mean ± SEM.

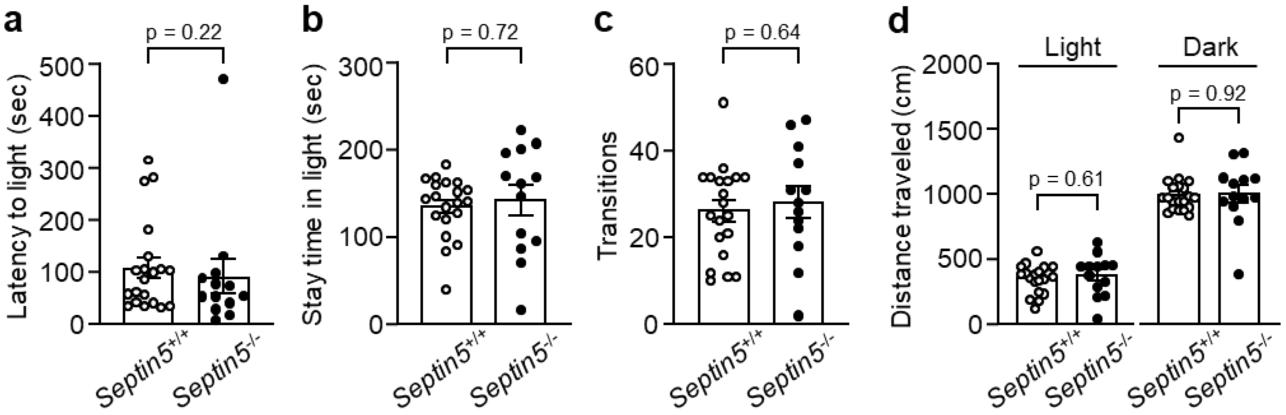

Figure S3

Light/dark transition test.

a, Latency until the first entry into the light chamber. **b**, Time spent in the light chamber. **c**, Number of transitions across the light/dark border. **d**, Distance traveled in the light and dark chambers. n = 20 (*Septin5*$^{+/+}$) and n = 13 (*Septin5*$^{-/-}$) 12–19-week-old male mice; normality was assessed with the Shapiro–Wilk test. When $p > 0.05$, variances were compared with the F test and, if equal, a two-tailed unpaired *t* test was used; if unequal, Welch's *t* test was used. When normality was rejected ($p < 0.05$), the Mann–Whitney test was used. Variance patterns were also inspected with a homoscedasticity plot. Mann–Whitney (**a**); Welch's *t* test (**b**, **d** dark); two-tailed unpaired *t* test (**c**, **d** light). Data are mean ± SEM.
